# 2.B. Round table: Rethinking innovation systems to meet the challenges of AMR: Lessons from the Covid-19 pandemic

**DOI:** 10.1093/eurpub/ckac129.066

**Published:** 2022-10-25

**Authors:** Organised by: RAND Europe and the European Observatory on Health Systems and Policy, Chair persons: Sonja Marjanovic (UK), Sarah Parkinson (UK)

## Abstract

Innovations have always been essential to solving our most pressing public health issues - from diagnostics to vaccines, to various therapeutics including antimicrobials. The ability of innovations to improve public health depends on the wider healthcare innovation systems that frame their development, uptake and diffusion. These innovation systems are made up of diverse actors including researchers and innovators in the public sector and industry, regulators, international bodies, not-for-profits, investors, healthcare professionals and patients and the public. However, the needs and interests of these diverse actors do not always align, nor do innovations systems always align with societal needs, particularly where there are market failures. Antimicrobial resistance (AMR) is an example of a public health issue that is challenging to solve within existing innovation systems, as incentive structures for developing and (not inappropriately) using antimicrobials are not sufficient to address the threat of growing resistance. In this workshop, we will facilitate a discussion of how innovation systems can be rethought to better align with societal needs, with a specific focus on how learning from the Covid-19 pandemic may be applied to innovation in response to the “silent pandemic” of AMR. This type of discussion matters greatly for tackling one of the most pressing public health challenges of our time and for harnessing the potential for timely learning and action. This panel discussion is co-hosted by RAND Europe and the European Observatory on Health Systems and Policy. Researchers from RAND Europe will first introduce the session and present key insights from their recent research on AMR-relevant innovation and lessons from the Covid-19 pandemic. This will provide the foundation for a panel discussion in which panelists will share their expertise and views on what lessons from the Covid-19 response mean for AMR and how these lessons can be feasibly applied to AMR-relevant innovation. Some areas for reflection include economic push and pull incentives, regulation, surveillance, data-sharing and data linkage, public-private collaboration, awareness raising and political will. Throughout the session, we will encourage participation from the audience and will ask participants to identify relevant questions in small groups at the beginning of the session. In bringing together diverse voices (on the panel and in the audience) that approach the innovation system from different angles, we hope to build an understanding of how the innovation system and mechanisms by which it operates can work better for everyone and tackle the urgent societal challenge of AMR, as well as explore whether any of the insights gained may apply more widely to other pressing public health challenges where there are market failures.

**Key messages:**

• Participants will gain a better understanding of how aspects of the Covid-19 response may be transferrable or adaptable to AMR-relevant innovation and how this can be achieved in a timely manner.

• Participants will identify needs for further research, policy and action to support innovation systems that are more responsive to AMR needs and potentially also to other public health challenges.

**Speakers/ Panellists:**

**Sonja Marjanovic**

RAND Europe, Cambridge, UK

**Sarah Parkinson**

RAND Europe, Cambridge, UK

**Nick Fahy**

RAND Europe, Cambridge, UK

**Dimitra Panteli**

European Observatory on Health Systems and Policies, Brussels, Belgium

**Michael Anderson**

London School of Economics and Political Science, Birmingham, UK

